# Ginsenoside CK Inhibits Hypoxia-Induced Epithelial–Mesenchymal Transformation through the HIF-1α/NF-κB Feedback Pathway in Hepatocellular Carcinoma

**DOI:** 10.3390/foods10061195

**Published:** 2021-05-26

**Authors:** Jingjing Zhang, Xiaoxuan Ma, Daidi Fan

**Affiliations:** 1 Shaanxi Key Laboratory of Degradable Biomedical Materials, School of Chemical Engineering, Northwest University, 229 Taibai North Road, Xi’an 710069, China; zhangjingjing@stumail.nwu.edu.cn (J.Z.); xiaoxuanma@163.com (X.M.); 2 Shaanxi R&D Center of Biomaterials and Fermentation Engineering, School of Chemical Engineering, Northwest University, 229 Taibai North Road, Xi’an 710069, China; 3 Biotech. & Biomed. Research Institute, Northwest University, 229 Taibai North Road, Xi’an 710069, China

**Keywords:** hepatocellular carcinoma, epithelial–mesenchymal transformation, hypoxia, NF-κB signaling, ginsenoside CK

## Abstract

Hepatocellular carcinoma (HCC) is a kind of malignant tumor with high morbidity and mortality rates worldwide. Epithelial–mesenchymal transformation (EMT) is crucial for HCC progression and prognosis. Characteristics of the tumor microenvironment, such as hypoxia, and excessive activation of the NF-κB signaling pathway have been identified as the key inducers of EMT in HCC. In our study, we verified the crosstalk between HIF-1α signaling and NF-κB pathway and their effects on EMT in HCC cells. The results show that CoCl_2_-induced hypoxia could promote IκB phosphorylation to activate NF-κB signaling and vice versa. Moreover, we found that ginsenoside CK, a metabolite of protopanaxadiol saponins, could inhibit the proliferation and colony formation of different HCC cell lines. Furthermore, ginsenoside CK could impair the metastatic potential of HCC cell lines under hypoxic conditions. Mechanistically, ginsenoside CK suppressed HIF-1α/NF-κB signaling and expression level of EMT-related proteins and cytokines in hypoxia-induced or TNFα-stimulated HCC cell lines. An in vivo study revealed that the oral delivery of ginsenoside CK could inhibit the growth of xenograft tumors and block HIF-1α and NF-κB signaling as well as EMT marker expression. Our study suggests that ginsenoside CK is a potential therapy for HCC patients that functions by targeting the HIF-1α/NF-κB crosstalk.

## 1. Introduction

Hepatocellular carcinoma (HCC) is a malignant tumor that accounts for 75–85% of the total cases of primary liver cancer [1] and is one of the leading causes of cancer-related death worldwide [2]. The morbidity and mortality rates of HCC have remained on an upward trend over the past two decades in the USA [3]. Early-stage HCC can achieve a relatively high cure rate through surgical resection, liver transplantation, radiofrequency ablation and other treatment methods; however, most patients are diagnosed with advanced hepatocarcinoma, which has poor treatment efficacy and survival quality. There are multiple factors affecting the therapeutic efficacy for HCC, including epithelial–mesenchymal transformation (EMT), which plays a crucial part in HCC progression and is characterized by the reducing expression of E-cadherin (epithelial marker) and increase in N-cadherin (mesenchymal marker) [4]. Through EMT, tumor cells not only have increased migration and invasion abilities but also prevent themselves from undergoing apoptosis and escape surveillance of the host immune system and drug therapy [5].

Among the various regulatory mechanisms of EMT, characteristics of the tumor microenvironment, such as hypoxia and low pH, are closely involved in EMT progression [6]. The rapid growth of tumor tissue often leads to oxygen starvation and necrosis in solid tumor cores because of the deficient blood supply caused by relatively slow angiogenesis [7]. When tumor cell proliferation is inhibited under hypoxia, multiple signaling pathways will be initiated for tumor cells to adapt to a hypoxic environment to ensure tumor development. Hypoxia is a common condition of malignant solid tumors, which may cause tumor necrosis while transforming the phenotype and gene expression of tumor cells to maintain proliferation. Studies have shown that, compared to patients with higher oxygen content, patients with lower oxygen content are not only more tolerant to radiation but also much more likely to relapse [8]. Hepatocellular carcinoma, with a median oxygen level of only 0.8% (compared with 3.9% in the normal liver) [9], is regarded as one of the most hypoxic tumors. Physiological processes have been developed to regulate cellular adaption to hypoxia, including hypoxia inducible factor-1α (HIF-1α), the primary response to hypoxia in most tissues. Under normoxia, HIF-1α can be hydroxylated by PHD, enabling binding with pVHL, which will lead to the further degradation of HIF-1α by E3 ubiquitin ligase [10]. In contrast, under hypoxia, the PHD activity was inhibited, resulting in the accumulation and nuclear translocation of HIF-1α, which could promote the transcriptional expression of hundreds of genes, including VEGF [11], and initiate numerous signaling pathways, such as the MAPK pathway [12]. HIF-1α stimulates EMT in tumors by regulating EMT-related signaling pathways (TGF-β, Notch, and Wnt/β-catenin signaling pathways) [13,14,15], modulating EMT-TF expression [16,17] and EMT-associated miRNA and lncRNA networks [18].

The nuclear factor-kappa B (NF-κB) signaling pathway, which can be triggered by tumor necrosis factor-alpha (TNFα), growth factors, ultraviolet radiation and some cytokines [19], is recognized in the EMT marker expression. The NF-κB subunit, composed of p65 (RelA) and p50, typically presents as an inactive form restricted to IκBα. Under the control of stimulating factors, IκBα is phosphorylated by the IKK complex and further degraded by the proteasome, leading to the release and nuclear translocation of the p65/p50 heterodimer, which contribute to the binding to DNA and the regulation of the transcription of numerous target genes, including various invasiveness-related genes, such as MMPs, intercellular adhesion molecule 1 (ICAM1), urokinase-type plasminogen activator (uPA) and so on [20]. The continuous activation of NF-κB has been confirmed to be necessary for the progression of hepatotoxicity and HCC [21], and targeting NF-κB is considered to be an effective therapeutic method for HCC.

Early research has illustrated the relation between HIF-1α and NF-κB in cancer development and progression [22]. HIF-1α and NF-κB signaling have been verified to individually induce EMT in several kinds of cancers [23,24,25]. However, further investigation is still needed about the crosstalk between HIF-1α and NF-κB and its effect on EMT in hepatocellular carcinoma cells.

Ginsenoside CK, metabolized by intestinal bacteria from other forms of orally administered protopanaxadiol saponins (PPDs), is a tetracyclic dammarane-type triterpenoid saponin [26]. As the major active player in ginsenoside efficacy, ginsenoside CK has better absorption in vivo and diverse pharmacological activities, such as anti-allergy, anti-inflammatory, antidiabetic, anti-cytotoxic, antiproliferative, anti-angiogenic and anti-invasion effects on lung, colorectal, gastric and brain tumors [27]. However, the effects on EMT in HCC mediated by the HIF-1α/NF-κB signaling pathways have not been reported yet.

In our experiment, we investigated the interaction effect between HIF-1α and NF-κB signaling and the contribution to EMT in HCC. Moreover, the ability of noncytotoxic concentrations of ginsenoside CK to inhibit the metastasis, invasion and EMT of HCC cells was estimated in vitro and in vivo. Our experimental data indicate that ginsenoside CK may be a potential treatment for HCC and that ginsenoside CK may have additional research value.

## 2. Materials and Methods

### 2.1. Chemicals and Reagents

Ginsenoside CK (Lot#: BP0651) was obtained from Chengdu Biopurify Phytochemicals Ltd. (Sichuan, China). Cell culture media (DMEM (Lot#: SH30243.01) and RPMI 1640 (Lot#: SH30809.01)) were purchased from HyClone (Logan, UT, USA). MTT (Lot#: ST316), DMSO (Lot#: ST038) and RIPA lysis buffer (Lot#: P0013B) were purchased from Beyotime Biotechnology (Shanghai, China). CoCl_2_ (Lot#: C804815) was obtained from Macklin Biochemical Co., Ltd. (Shanghai, China). Trypsin (Lot#: T8150), penicillin/ streptomycin (Lot#: P1400), crystal violet (Lot#: G1063), PMSF (Lot#: P0100), phosphatase inhibitor cocktail (Lot#: P1260) and BCA protein assay reagent kits (Lot#: PC0020) were purchased from Solarbio Science & Technology Co., Ltd. (Beijing, China). FBS (Lot#: 04-001-1ACS) was purchased from Biological Industries (Kibbutz Beit Haemek, Israel). Rabbit antibodies against E-cadherin, Vimentin, N-cadherin, Snail, HIF-1α, Twist1, IκB-α, p65, Lamin B1, ICAM-1, CA9 and c-Myc and mouse antibodies against HIF-1α and VEGF were obtained from Proteintech Group, Inc (Chicago, IL, USA). Anti-phospho-IκB-α and anti-phospho-p65 rabbit antibodies were purchased from Cell Signaling Technology (Boston, MA, USA), and anti-MMP-2 and anti-MMP-9 rabbit antibodies were purchased from Abways Technology (Shanghai, China). Details about antibodies are shown in Table S1.

### 2.2. Cell Lines and Cell Culture

The HCC cell lines HepG2, SMMC-7721, HCC-LM3, Huh-7 and HLE were purchased from the Shanghai Institute of Cell Biology. Normal liver cell line LX2 was obtained from Shanghai Gaining Biological Technology Co., Ltd. HepG2, Huh-7, HLE, HCC-LM3 and LX2 cells were incubated in DMEM supplemented with 10% (*v*/*v*) FBS, 100 units/mL penicillin and 100 mg/mL streptomycin. SMMC-7721 cells were cultured in RPMI-1640 medium with 10% (*v*/*v*) FBS supplemented with 100 units/mL penicillin and 100 mg/mL streptomycin. Cells were maintained at 37 °C in a humidified incubator with 5% CO_2_ in air.

### 2.3. In Vitro Cytotoxicity Assay

In vitro cytotoxicity was measured by an MTT assay. HepG2, Huh-7, SMMC-7721, HCC-LM3, HLE and LX2 cells were seeded in 96-well plates at a density of 1 × 10^4^ cells/well for 24 h. Afterwards, different concentrations of ginsenoside CK (2.5–100 μM) and CoCl_2_ (50–500 μM) were added. After incubating for an additional 48 h, MTT (0.5 mg/mL) was added and cultured for 2–4 h. Finally, MTT was removed carefully followed by addition of 150 μL DMSO to each well, and the plates were detected by a microplate reader (Bio-Tek Instruments, Inc., Winooski, VT, USA) at 490 nm. Each data point was an average of the values of six replicates at each concentration.

### 2.4. Cell Colony Forming Assay

HCC cells were distributed in 6-well plates (500 cells/well). After 8 h of attachment, the cells were treated with ginsenoside CK for 48 h. Then, the medium was substituted with drug-free medium every 2 days. Culture medium was removed after 14 days. The formed cell colonies were fixed with methanol and stained with 0.1% crystal violet. Colony formation was recorded by photographing. 

### 2.5. Flow Cytometry Analysis

Cells were seeded in 6-well plates and incubated for 24 h. Then, the cells were treated with ginsenoside CK for an additional 24 h. Subsequently, cells were collected by centrifugation followed by fixation with ice-cold 70% ethanol overnight. The fixed cells were treated with PBS containing 0.5 mg/mL RNase A and stained with 50 mg/mL PI for 30 min in the dark. The fluorescence intensity of individual nuclei was measured by flow cytometry, and the data were analyzed with FlowJo software.

### 2.6. Cell Migration and Invasion Assay

For the cell scratch test, HCC-LM3, SMMC-7721 and HepG2 cells were seeded in 6-well plates (1 × 10^6^ cells/well). After cell adherence, the formed cell monolayers were scratched with 10 μL pipette tips and washed with PBS to remove floating cells. Cells were incubated under normoxia or hypoxia (induced with 200 μM CoCl_2_) with or without ginsenoside CK (5 μM and 10 μM) for 36 h. The migration distance was calculated using IPP software. Three replicates were performed at each concentration.

For the transwell migration assay, HCC-LM3 cells (1 × 10^5^ cells/well) were seeded in the upper compartment of the transwell chamber with serum-free conditioned medium (8 μm pore size, Corning, NY, USA). Then, 0.6 mL culture medium with 10% FBS was added to the lower compartment. The plates were maintained for 24 h at 37 °C. After incubation, the filter was fixed with methanol and stained with 0.1% crystal violet. The cells on the upper surface of the filter were removed, and cells on the lower surface were photographed under a microscope at 100× magnification followed by dissolution with 30% acetic acid. The solution was measured using a wavelength of 592 nm.

The method of the cell invasion assay was the same as that of the cell migration assay except that the upper chamber was precoated with 50 μg of matrigel (Corning, NK, USA).

### 2.7. Immunofluorescence

Cells treated with CoCl_2_, TNFα and ginsenoside CK were fixed with 4% paraformaldehyde and blocked with 10% BSA. The primary antibodies were added and treated for an additional 4 h, and then the CoraLite 594-conjugated secondary antibody was added and incubated for 1 h. DAPI was utilized to stain the cell nuclei. The fluorescent signals were captured using a laser scanning confocal microscope (Leica, Wetzlar, Germany).

### 2.8. RNA Isolation and RT-qPCR

After treatment with CoCl_2_, TNFα and ginsenoside CK, cells were lysed with TRIzol reagent, and total RNA was isolated. cDNA was synthesized using a RevertAid First Strand cDNA Synthesis Kit (Thermo Fisher Scientific, MA, USA), and PCR was performed with a qRT-PCR system (Bio-Rad, Hercules, CA, USA). The expression level of the GAPDH gene was used to normalize the relative RNA expression levels for each gene. Primer sequences used are listed in Table S2.

### 2.9. Animal Studies of Hepatocellular Carcinoma

Animal experiments were performed in accordance with the Institutional Animal Care and Use Committee guidelines. Male BALB/c nude mice were obtained from GemPharmatech Co., Ltd. (Jiangsu, China).

To assess the antitumor response of ginsenoside CK, 1 × 10^7^ HCC-LM3 cells in 200 μL FBS-free medium were subcutaneously injected into the left axilla of the mice. Once the tumor volume reached 100 mm^3^, the mice were divided into four groups (7 mice per group) and orally gavaged with the vehicle control (mixture of poloxamer (14.63%), PEG 400 (40.18%), propylene glycol (40.19%) and ethyl alcohol (5%)), ginsenoside CK (30, 60 mg/kg) and sorafenib (60 mg/kg) every day for 6 weeks. The tumor volume and body weight were measured each week. At the end of the experiments the mice were sacrificed, and the primary tumors and some major organs were collected and weighed.

### 2.10. Western Blotting

Protein samples were extracted from nuclei, whole cells and tissues. The total protein content was quantified using a BCA protein assay kit (Solarbio, Beijing, China). Samples containing the same amounts of protein were subjected to SDS-PAGE and electrotransferred to PVDF membranes (Millipore, Bedford, MA, USA). The membranes were then blocked with dried nonfat milk in TBST buffer for 2 h, followed by binding with the primary antibodies at 4 °C overnight. After washing with TBST for 5 times, the membranes were probed with HRP-conjugated secondary antibodies for an additional 1 h. The antigen–antibody complexes were visualized with an ECL chemiluminescence kit. GAPDH and Lamin B1 were used as internal controls to verify protein loading.

### 2.11. Immunohistochemistry Assay

The xenograft tumor tissues and other main organ samples were fixed with formalin. Partial tumors and organs were performed with H&E staining. The tumor specimens were also stained with immunohistochemistry for p53, HIF-1α, *N*-cadherin, phospho-IκB-α, IκB-α and Vimentin. Images were captured with a light microscope (Leica, Germany).

### 2.12. Statistical Analysis

The experimental data were analyzed with SPSS and GraphPad Prism software using one-way analysis of variance (ANOVA), and a *p* value < 0.05 was considered statistically significant. The data are shown as the mean ± standard deviation (SD).

## 3. Results

### 3.1. Transcription of HIF-1α in Cancers and the Correlation with NF-κB Signaling

**Figure 1:**
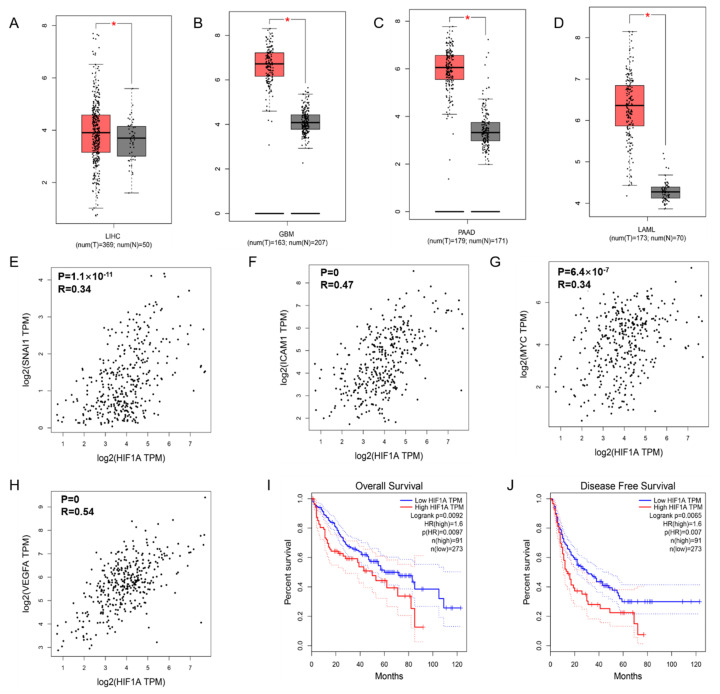
HIF-1α and NF-κB signaling related gene expression and the effect on prognosis in HCC. HIF-1α expression in liver hepatocellular carcinoma (LIHC) (**A**), glioblastoma multiforme (GBA) (**B**), pancreatic adenocarcinoma (PAAD) (**C**), and acute myeloid leukemia (LAML) (**D**) compared with normal group based on TCGA and GTEx dataset. The positive relationship between the expression of HIF-1α and EMT-related cytokine Snail1 (**E**), the NF-κB targeted genes ICAM-1 (**F**), c-Myc (**G**), and VEGFA (**H**). The overall survival curve (**I**) and disease-free survival curve (**J**) of patients with HCC according to HIF-1α gene expression are presented. The *p* value is affirmed by log-rank test. The Cox proportional hazard ratio and the 95% confidence interval information are included. * *p* < 0.05 vs. normal group.

To confirm the expression of HIF-1α in tumors, we searched and analyzed the data in the GEPIA database and found that HIF-1α was more highly expressed in liver hepatocellular carcinoma (LIHC) (Figure 1A), glioblastoma multiforme (GBM) (Figure 1B), pancreatic adenocarcinoma (PAAD) (Figure 1C) and acute myeloid leukemia (LAML) (Figure 1D) tissues than in healthy tissues. Moreover, the HIF-1α expression level was related to the length of overall survival (Figure 1I) and disease-free survival (Figure 1J) of HCC patients, where high HIF-1α expression corresponded to poor survival outcomes. In addition, we observed a positive association between HIF-1α and the EMT-related cytokine Snail (R = 0.34, *p* = 1.1 × 10^−11^; Figure 1E), HIF-1α and the NF-κB target gene ICAM-1 (R = 0.47, *p* = 0; Figure 1F), HIF-1α and c-Myc (R = 0.26, *p* = 6.4 × 10^−7^; Figure 1G), and HIF-1α and VEGFA (R = 0.54, *p* = 0; Figure 1H), which suggests the potential correlations among HIF-1α, NF-κB and EMT in HCC.

### 3.2. Ginsenoside CK Inhibits the Viability and Proliferation Characteristics of HCC Cells

**Figure 2:**
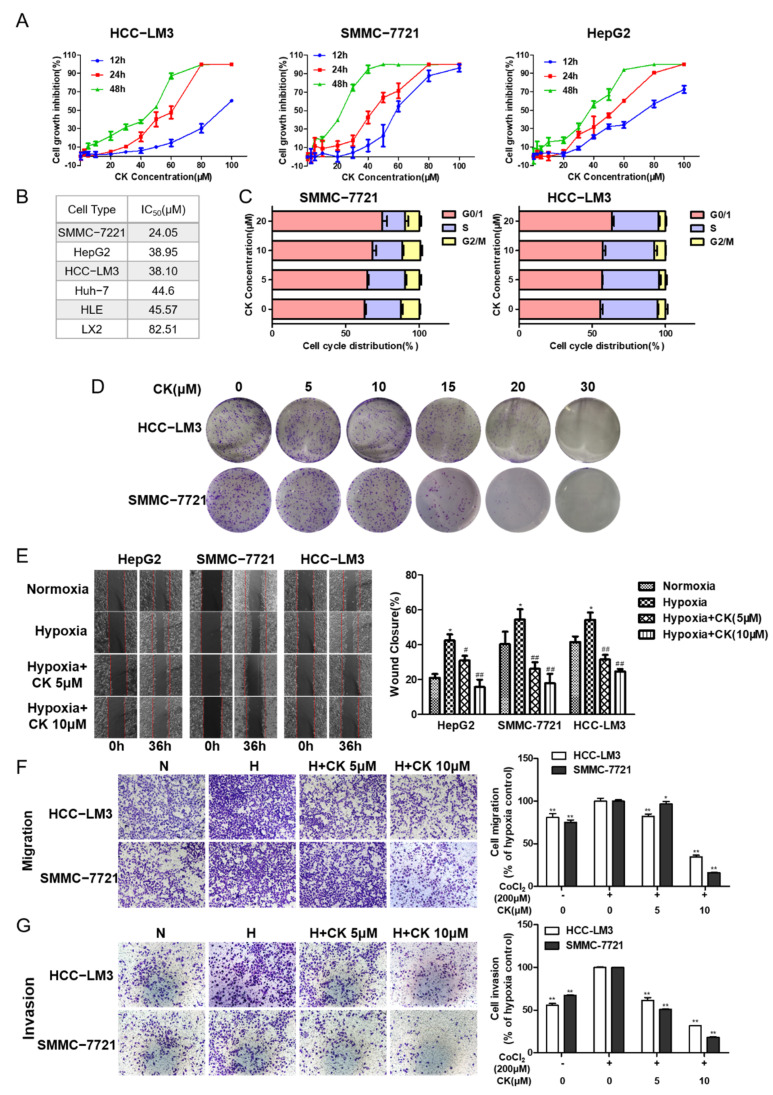
Ginsenoside CK inhibits cell proliferation, colony formation and metastasis in HCC cells. (**A**) Cytotoxicity of ginsenoside CK treatment on HCC cell lines after 12 h to 48 h were determined by MTT assay. Figures are presented as mean ± SD of six replicates. (**B**) The IC50 value of ginsenoside CK on different HCC cell lines for 48 h. (**C**) Effect of ginsenoside CK on cell cycle distribution. HCC-LM3 and SMMC-7721 cells were treated with different concentration of CK (5, 10 and 20 μM) for 24 h. Cell cycle distribution was analyzed using PI staining assay. The data were presented as mean ± SD of three experiments. (**D**) Effect of ginsenoside CK on the colony formation of HCC-LM3 and SMMC-7721 cells. Representative images of colony formation were from one of three independent experiments. Effects of ginsenoside CK on metastasis potential of HCC cell lines were determined by scratch assay (**E**) and transwell assay (**F**,**G**). Cells were incubated under normoxia or hypoxia induced by CoCl_2_ and treated with different doses of CK. For the cell scratch assay, the width of wound region was evaluated 36 h after scratching by microscopy at 100× magnification. In the transwell assay, representative migratory and invaded cells were stained with crystal violet, and the data were presented as the percentage of CoCl_2_-treated cells. * *p* < 0.05, ** *p* < 0.01 vs. normoxia group. # *p* < 0.05, ## *p* < 0.01 vs. hypoxia group.

The cytotoxic effect of ginsenoside CK on different HCC cells was verified with an MTT assay. The data indicate that ginsenoside CK could suppress the proliferation of HCC cell lines (including HCC-LM3, HepG2 and SMMC-7721) in a time- and dose-dependent manner (Figure 2A). The IC50 values of ginsenoside CK in different HCC cell lines varied from 20 to 50 μM after treatment for 48 h (Figure 2B). In addition, the cytotoxic effect of ginsenoside CK on normal liver cells was less sensitive, as with much higher IC50 value for 48 h. Moreover, the cell cycle analysis data show that the cell cycles of HCC-LM3 and SMMC-7721 cells were arrested at the G0/1 phase after ginsenoside CK treatment (Figure 2C).

Next, we detected the effects of ginsenoside CK on the cellular multiplication capacity by a colony-forming assay. As shown in Figure 2D, colony formation after 5–15 μM ginsenoside CK treatment was not obviously impacted compared to colony formation after vehicle treatment. However, the proliferation ability was significantly decreased when the concentration of ginsenoside CK was greater than 15 μM in HCC-LM3 and SMMC-7721 cells.

### 3.3. Ginsenoside CK Suppresses Hypoxia-Induced Metastasis and Invasion of HCC Cells

The metastatic and invasive potentials of HCC cells were detected by wound healing and transwell assays. CoCl_2_, which can inhibit hydroxylation modification and further degradation of HIF-1α, was used to mimic hypoxic conditions in vitro. In the wound scratch assay, cells were treated with CoCl_2_ (200 μM), which was verified to have little cytotoxicity against HCC cell lines (Figure S1), and ginsenoside CK at concentrations of 5 and 10 μM. The results demonstrate that, compared with normoxia cell culture conditions, hypoxia cell culture conditions significantly promoted cellular migration. This induction was restricted in a concentration-dependent manner after treatment with ginsenoside CK in three HCC cell lines (Figure 2E).

For the migration assay, HCC-LM3 and SMMC-7721 cells were selected according to lower IC50 values and their mesenchymal features. As shown in Figure 2F, the migration rate of HCC-LM3 and SMMC-7721 cells was accelerated by 23.76% and 32.90% compared with that of the control cells, upon treatment with CoCl_2_. Compared with CoCl_2_ treatment, treatment with ginsenoside CK inhibited the motility of the cells by 17.79–57.95% and 10.11–81.96% in a dose-dependent manner (* *p* < 0.05, ** *p* < 0.01).

For the matrigel transwell invasion assay, the invasion rates of HCC-LM3 and SMMC-7721 cells were 79.05% and 48.12%, respectively, higher under hypoxia than under normoxia (** *p* < 0.01). We further studied whether ginsenoside CK could inhibit the invasion induced by hypoxia. Compared with that of the CoCl_2_-only group, the invasion capacity of cells in the CoCl_2_ + ginsenoside CK group showed a concentration-dependent reduction of 38.40–48.34% in HCC-LM3 cells and 49.05–64.25% in SMMC-7721 cells (** *p* < 0.01) (Figure 2G), indicating the inhibitory effect of ginsenoside CK on the invasion ability.

### 3.4. Ginsenoside CK Inhibits HCC Tumor Growth In Vivo

**Figure 3:**
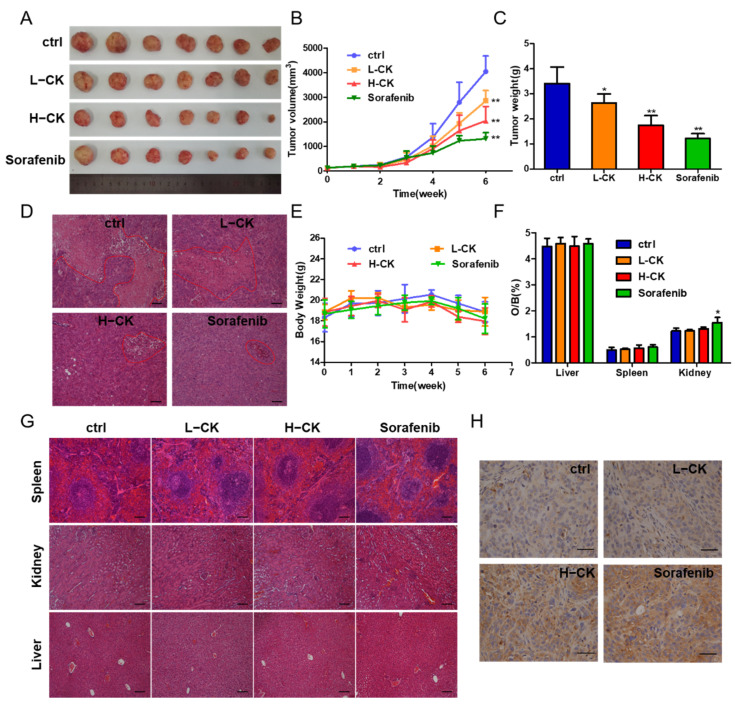
Ginsenoside CK inhibited the growth of HCC cell xenografts in vivo. (**A**) Photograph of tumors from four groups (control group, CK treated group and sorafenib treated group). Sorafenib was used as positive control. (**B**) Tumor volume of the xenograft models was measured every week during the experiments. (**C**) Tumor weight of the xenograft models at the end of the experiment. (**D**) HE staining showing tumor necrosis (marked with red lines). Magnification, 100×. (**E**) Body weight of the nude mice was measured every week during the experiment. (**F**) Ratio of weight of main organs to body weight of mice. (**G**) HE staining showing the main organs of xenograft models, magnification 100×. (**H**) Photographs of p53 immunohistochemical (IHC) stained tumor sections, magnification 400×. All quantitative data were represented as mean ± SD, n = 7. * *p* < 0.05, ** *p* < 0.01 vs. control group.

According to previous results, we confirmed that ginsenoside CK could inhibit cell migration and invasion in vitro. Furthermore, the antitumor activity of ginsenoside CK against HCC in vivo was tested in HCC-LM3 cell lines in nude mouse models. Compared with the control, ginsenoside CK treatment prominently suppressed tumor growth by diminishing the tumor volume and the tumor weight in a dose-dependent manner (Figure 3A–C) with little influence on the body weight and main organ weight of the mice as shown in Figure 3E,F. The inhibition rate of the group treated with 60 mg/kg ginsenoside CK reached 54.35%, while the rate of sorafenib reached 67.64% as the positive control group. The specimens also displayed smaller necrotic areas in the ginsenoside CK-treated group than in the vehicle-treated group (Figure 3D). Moreover, H&E staining of the main organs, including the liver, kidney and spleen, showed that in addition to the slight inflammatory response in the kidney caused by sorafenib, which corresponds to the change in kidney weight, the other specimens all seemed normal compared with the control group (Figure 3F,G). In addition, p53 staining in the xenograft models demonstrated that ginsenoside CK could suppress the proliferation of HCC cells in vivo (Figure 3H).

### 3.5. Crosstalk between HIF-1α and NF-κB Signaling in HCC Cells

**Figure 4:**
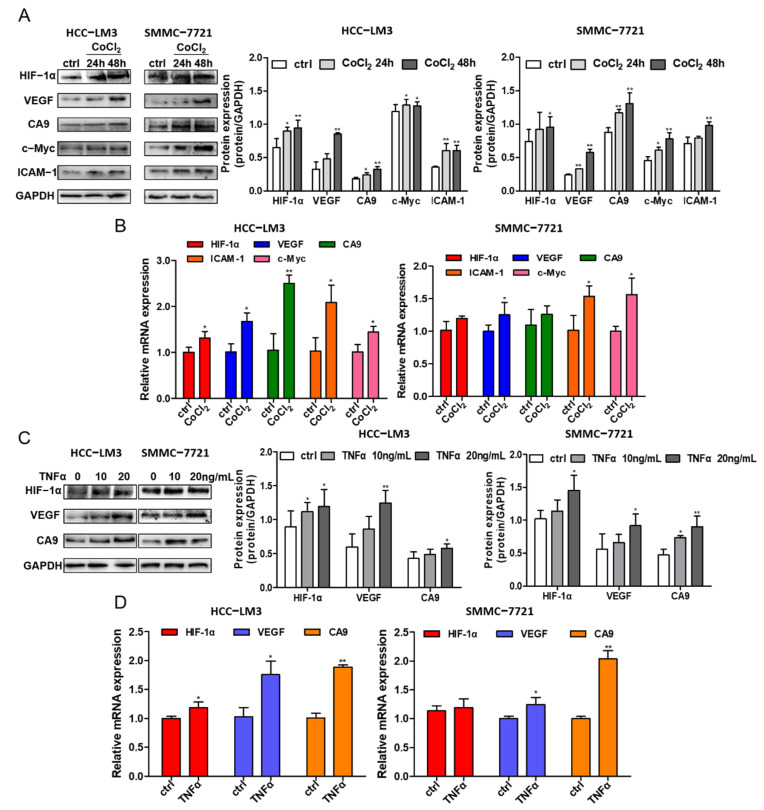
The crosstalk between HIF-1α and NF-κB signaling pathway. (**A**) HCC-LM3 and SMMC-7721 cells were treated with 200 μM CoCl_2_ for 24 h and 48 h. HIF-1α, the targeted protein CA9, VEGF, NF-κB signaling downstream gene c-Myc and ICAM-1 were assessed by Western blotting. Quantification plots are shown on the right. (**B**) HCC-LM3 and SMMC-7721 cells were treated with 200 μM CoCl_2_ for 24 h; HIF-1α, CA9, VEGF, cMyc and ICAM-1 mRNA levels were detected by RT-PCR. Gene expression is normalized to GAPDH. The data were presented as mean ± SD, * *p* < 0.05, ** *p* < 0.01 compared with untreated cells. (**C**) HCC-LM3 and SMMC-7721 cells were induced with 10 ng/mL and 20 ng/mL TNFα for 24 h; HIF-1α, CA9 and VEGF protein expression were determined by Western blotting. Quantification plots are shown on the right. (**D**) HCC-LM3 and SMMC-7721 cells were induced with 20 ng/mL TNFα for 24 h; HIF-1α, CA9 and VEGF mRNA expression values were assessed by RT-PCR. Gene expression is normalized to GAPDH. The data were presented as mean ± SD, * *p* < 0.05, ** *p* < 0.01 compared with control group. Data are representative of three independent experiments.

Hypoxic conditions were induced in different HCC cell lines (HCC-LM3 and SMMC-7721) at different times (24 h and 48 h) by 200 μM CoCl_2_. The protein expression levels of HIF-1α; the HIF-1α target genes VEGF and CA9; the NF-κB downstream signaling protein ICAM-1; and c-Myc were assessed by Western blotting, and simultaneously the mRNA of HIF-1α, CA9, VEGF, cMyc and ICAM-1 were evaluated by RT-qPCR. Under CoCl_2_ activation, the protein level of HIF-1α was enhanced, while the HIF-1α gene level had little change (Figure 4A,B), which is in accordance with the theory of hypoxia induced by CoCl_2_. The increase in the transcriptional expression levels of CA9 and VEGF in HCC cell lines indicate the activation of HIF-1α signaling (Figure 4A,B). In addition, the gene and protein expressions of ICAM-1 and c-Myc were improved (Figure 4A), implying the possible acceleration of the NF-κB signaling pathway under hypoxic conditions.

Treating HCC cell lines with 10 ng/mL and 20 ng/mL TNFα, a stimulating factor of NF-κB signaling, increased the protein level of HIF-1α and its targeted proteins VEGF and CA9, as shown by Western blotting analysis in two HCC cell lines (Figure 4C). The gene expression levels of HIF-1α, VEGF and CA9 were also detected, and there was an increase in VEGF and CA9 after treatment with TNFα in the HCC-LM3 and SMMC-7721 cell lines, while the gene level of HIF-1α fluctuated (Figure 4D). In summary, the presented data suggest that HIF-1α stabilization induced under hypoxic conditions could activate NF-κB signaling, which may further regulate the expression of HIF-1α, demonstrating the crosstalk between HIF-1α and the NF-κB signaling pathway.

### 3.6. Hypoxia and NF-κB Signaling Stimulate EMT Progression in HCC Cells

**Figure 5:**
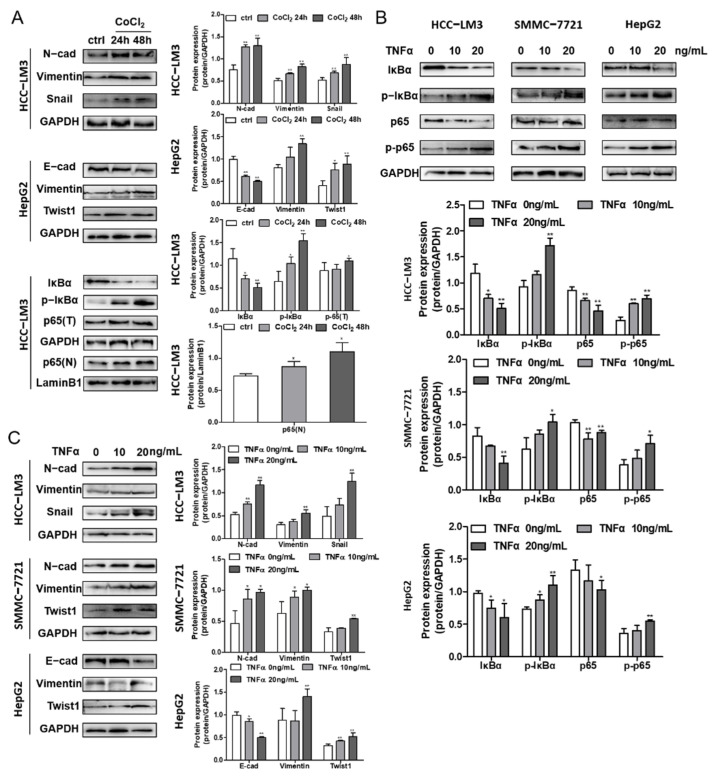
The effects of hypoxia and the NF-κB signaling pathway on EMT in HCC cells. (**A**) EMT marker protein and cytokines expression in CoCl_2_ induced HCC-LM3 and HepG2 cell lines were analyzed by Western blotting. IκBα, p-IκBα, total p65 (p65(T)) and nucleus p65 (p65(N)) expression levels in CoCl_2_ stimulated HCC-LM3 cells were assessed. Quantification plots are presented on the right. GAPDH and Lamin B1 were used as control proteins. (**B**) HCC-LM3, SMMC-7721 and HepG2 cells were stimulated with 0, 10, and 20 ng/mL TNFα. IκBα, p-IκBα, p65 and p-p65 expressions were assessed by Western blot analysis. Quantification plots are shown below. (**C**) EMT maker protein and cytokines expression level were detected by Western blotting in TNFα-treated HCC-LM3, SMMC-7721 and HepG2 cells. Quantification plots are shown on the right. Data were shown as mean ± SD values (n = 3). * *p* < 0.05, ** *p* < 0.01 compared with untreated control cells. The figures shown were representative of three independent experiments.

HCC-LM3, SMMC-7721 and HCC-LM3 cells were incubated with 200 μM CoCl_2_ for 24 and 48 h, and the expression levels of proteins related to the NF-κB pathway and EMT were determined. The data illustrate the decrease in IκBα and the promotion of p-IκBα, which contributed to the release and nuclear translocation of the p65/p50 subunit, revealing the activation of the NF-κB pathway (Figure 5A), further confirming the interaction of HIF-1α and NF-κB signaling. Moreover, TNFα-induced cells showed the same IκBα and p-IκBα protein levels, illustrating the increase in NF-κB signaling (Figure 5B). Along with the irradiation of HIF-1α and NF-κB, EMT marker proteins were assessed to determine the influence of signaling on EMT in HCC cells. The downregulation of E-cadherin and upregulation of N-cadherin, Vimentin, and the cytokines Snail and Twist1 suggested that EMT was induced by NF-κB and hypoxia in the three cell lines (Figure 5A,C).

### 3.7. Ginsenoside CK Reversed Hypoxia-Induced EMT in HCC Cells

**Figure 6:**
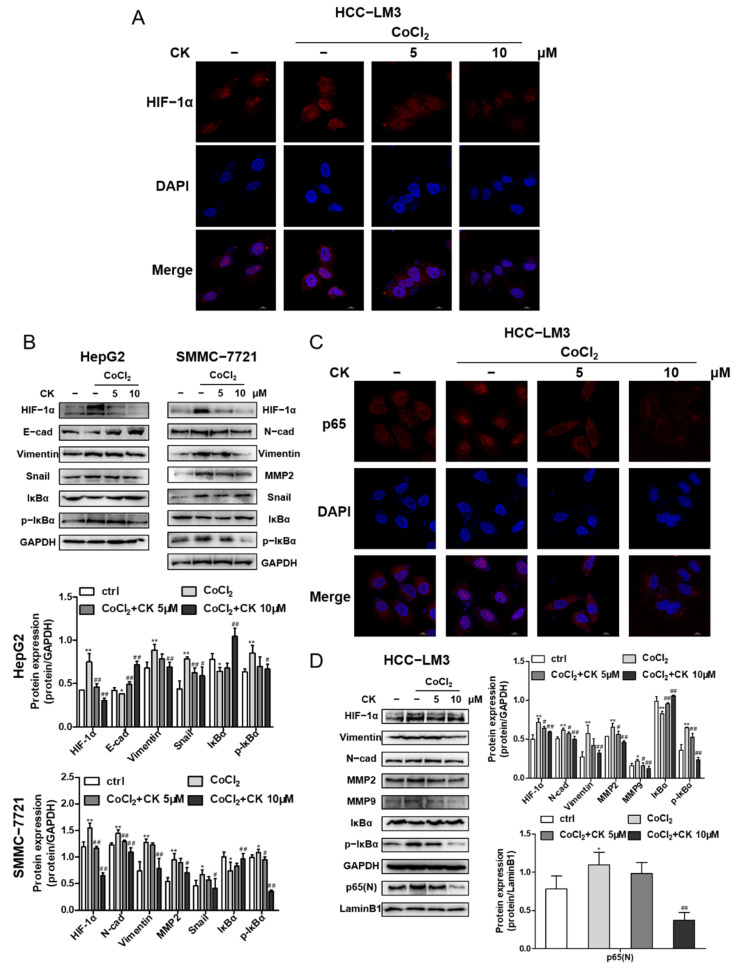
Ginsenoside CK inhibited hypoxia induced EMT in vitro. (**A**) HCC-LM3 cells were treated with ginsenoside CK in the absence or presence of 200 μM CoCl_2_ for 24 h. HIF-1α (red), DAPI (blue) immunofluorescence staining, and 2-channel merged images showed the protein expression level and the nuclear translocation of HIF-1α. Magnification 1000×. (**B**) HepG2 and SMMC-7721 cells were treated with ginsenoside CK in the absence or presence of 200 μM CoCl_2_ for 24 h. HIF-1α, EMT related proteins, IκBα and p-IκBα expressions were determined by Western blotting. Quantification plots are shown below. Data were presented as mean ± SD (n = 3). HCC-LM3 cells were treated with ginsenoside CK in the absence or presence of 200 μM CoCl_2_ for 24 h. (**C**) The immunofluorescence staining of p65 (red) and 2-channel merged images showed the nuclear translocation of p65. Magnification 1000×. (**D**) HIF-1α, EMT related proteins, MMP2, MMP9, IκBα, p-IκBα and nuclear p65 proteins were assessed by Western blot analysis. Quantification plots are shown on the right. Data were presented as mean ± SD (n =3). * *p* < 0.05, ** *p* < 0.01 compared with untreated control cells. # *p* < 0.05, ## *p* < 0.01 vs. CoCl_2_-treated group. The figures shown were representative of three independent experiments.

Immunofluorescence staining demonstrated that ginsenoside CK effectively inhibited the expression and translocation of HIF-1α induced by hypoxia in HCC-LM3 cells, as shown in Figure 6A, which corresponds to the Western blotting results shown in Figure 6D. Next, we detected the influence of ginsenoside CK on HIF-1α downstream signaling. HCC cells incubated with CoCl_2_ and treated with ginsenoside CK not only downregulated the HIF-1α-targeted proteins but also reversed the expression level of the hypoxia-induced EMT markers E-cadherin, N-cadherin, Vimentin, Snail and matrix metalloproteinases (MMP2 and MMP9) (Figure 6B,D). Moreover, we found that ginsenoside CK inhibited the IκB phosphorylation stimulated under hypoxia in the three cell lines (Figure 6B,D). Subsequently, the nuclear translocation of p65 was also assessed by immunofluorescence staining. The data show that the nuclear translocation of p65 was promoted under hypoxia, which further confirmed the relationship between HIF-1α and NF-κB signaling, and this increase could be reversed by ginsenoside CK in a concentration-dependent manner (Figure 6C). The immunoblotting data are shown in Figure 6D.

### 3.8. Ginsenoside CK Suppresses TNF-α-Induced EMT of HCC In Vitro

We assessed whether ginsenoside CK could suppress TNFα-induced migration and invasion by wound scratching. In the wound healing assay, SMMC-7721 cell migration accelerated by TNFα was obviously reduced with ginsenoside CK treatment, as shown in Figure S2.

**Figure 7:**
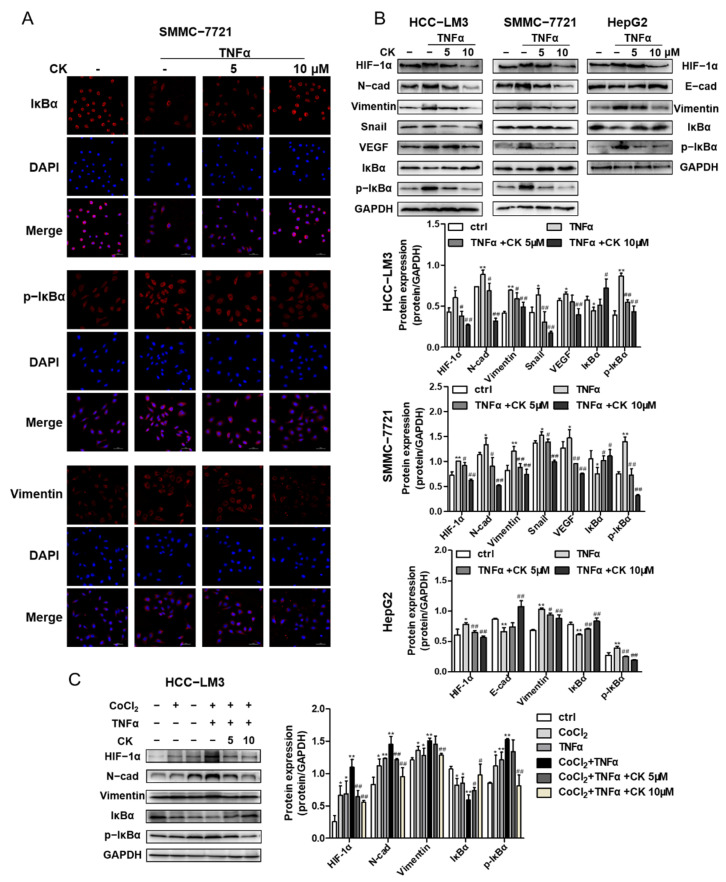
Ginsenoside CK suppressed TNFα-stimulated EMT of HCC in vitro. (**A**) HCC-LM3, SMMC-7721 and HepG2 cell lines were treated with 5 and 10 μM CK in the absence or presence of 20 ng/mL TNFα for 24 h. HIF-1α, EMT related proteins and cytokines, IκBα and p-IκBα were assessed by Western blot analysis. (**B**) SMMC-7721 cells were treated with 5 and 10 μM CK in the absence or presence of 20 ng/mL TNFα for 24 h. The immunofluorescence staining of IκBα (red), p-IκBα (red) and vimentin (red) is presented. DAPI (blue) was used to mark the cell nuclear. Magnification 400×. (**C**) HCC-LM3 cells were treated with ginsenoside CK in the absence or presence of CoCl_2_ and TNFα. HIF-1α, EMT related proteins, IκBα and p-IκBα were assessed by Western blot analysis. Quantification plots are shown below. Data were shown as mean ± SD (n = 3). * *p* < 0.05, ** *p* < 0.01 compared with untreated control cells. # *p* < 0.05, ## *p* < 0.01 vs. TNFα-treated group. The figures shown were representative of three independent experiments.

To determine whether ginsenoside CK could inhibit epithelial–mesenchymal transition monitored through the NF-κB signaling pathway, the transcriptional expression of EMT marker proteins, EMT transcription factors, IκBα and p-IκBα was detected by an immunoblotting assay and immunofluorescence staining. The results demonstrate that ginsenoside CK reduced N-cadherin, Vimentin and Snail expression in HCC-LM3, SMMC-7721 and HepG2 cells, as well as the phosphorylation of IκB induced by TNFα (Figure 7B). The staining of Vimentin, IκBα and p-IκBα in SMMC-7721 cells also accounted for the inhibitory activity of ginsenoside CK (Figure 7A). In addition, Western blot analysis revealed that ginsenoside CK also blocked the HIF-1α and VEGF expressions enhanced by TNFα (Figure 7B), suggesting that ginsenoside CK intercepted the crosstalk between HIF-1α and NF-κB.

In addition, we performed experiments with HCC-LM3 cells that were first treated with CoCl_2_ and then activated by TNFα to study the effects of hypoxia and TNFα on HCC cells. The results of Western blotting showed TNFα also could enhance the protein level of HIF-1α under hypoxia conditions as well as EMT marker proteins (N-cad and Vimentin). Changes to the level of IκBα and p-IκBα indicated the synergistic effect of TNFα and CoCl_2_, which could be reversed with ginsenoside CK (Figure 7C).

### 3.9. Ginsenoside CK Blocks the EMT of HCC In Vivo

**Figure 8:**
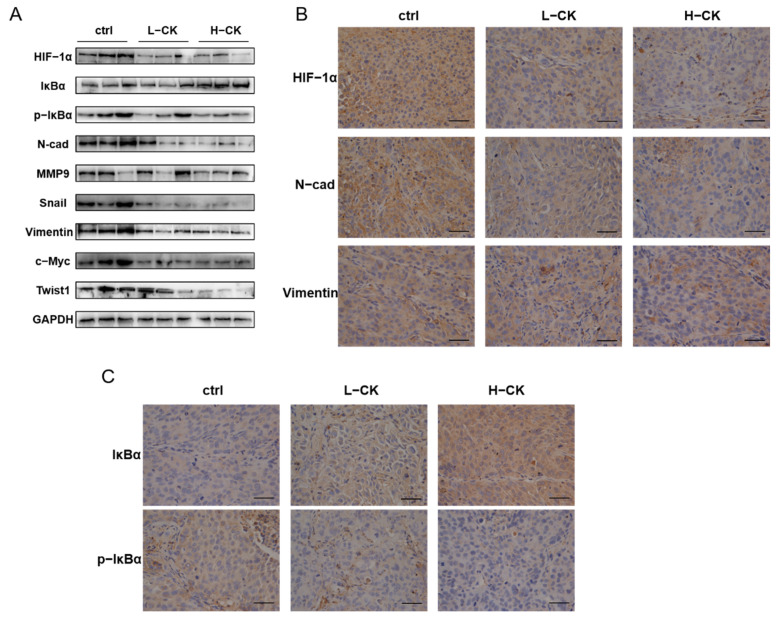
Ginsenoside CK blocked the EMT in HCC cells through suppressing the HIF-1α signaling and NF-κB signaling pathway in vivo. (**A**) Western blotting of HIF-1α, EMT markers, cytokines, metastasis related protein and NF-κB signaling in HCC-LM3 xenograft tumor specimens. The results shown were representative of three independent experiments. (**B**) IHC staining of HIF-1α, *N*-cadherin and Vimentin in HCC-LM3 xenografts. Magnification 400×. (**C**) Tumor specimens from the xenograft were immunohistochemically stained with anti-IκBα and anti-p-IκBα antibody. Magnification 400×.

In HCC-LM3 xenograft models, tumor specimens were analyzed by H&E staining, immunohistochemistry and Western blot assays. Tumors treated with ginsenoside CK showed lower levels of hypoxia, as demonstrated by immunoblotting (Figure 8A) and immunohistochemistry staining (Figure 8B), implying that the accumulation and activity of HIF-1α in HCC-LM3 xenograft tumors can be regulated by treatment with ginsenoside CK. In addition, the phosphorylation of IκBα was subdued in xenograft tumors by ginsenoside CK, as well as EMT markers, such as *N*-cadherin and Vimentin, which is in accordance with the in vitro results (Figure 8B,C).

## 4. Discussion

In previous studies, ginsenoside CK has been demonstrated to have pleiotropic activity in various cancers [27]. Extensive mechanistic studies have shown that ginsenoside CK can induce apoptosis and cell cycle arrest, inhibit angiogenesis and sensitize human colon cancer, lung cancer, breast cancer, glioblastoma, liver cancer and more [28,29,30,31,32]. However, the inhibitory activities of ginsenoside CK on cancer metastasis have been less reported. In our present study, ginsenoside CK showed notable cytotoxicity to HCC cells with an IC50 of 20–50 μM for 48 h. In epithelium-like HCC cells (HepG2) and mesenchymal-like cells (SMMC-7721 and HCC-LM3), the proliferation potential was suppressed by ginsenoside CK in a concentration-dependent manner. In addition, ginsenoside CK arrested the cell cycles of SMMC-7721 and HCC-LM3 cells in the G0/G1 phase in a dose-dependent manner. Furthermore, the reduction in tumor volume and tumor weight verified the anticancer effects of ginsenoside CK in vivo. A previous study on the proliferation-inhibiting effects of ginsenoside CK on HepG2 cells showed the IC50 value of 24.9 μM [33], while a later study showed that the IC50 of ginsenoside CK on the same cell was only 11.4 μM [34]. Differences in the cytotoxicity of ginsenoside CK were also found in SMMC-7721 cells. The SMMC-7721 cell survival rates following treatment with 5 μM ginsenoside CK at 48 h remained nearly 30%. However, the cell cycle of SMMC-7721 cells was arrested at the G0/G1 phase upon treatment with ginsenoside CK [35], which is in accordance with our results. In addition, treatment with higher doses of ginsenoside CK induced cell cycle arrest and cytotoxicity, while lower concentrations exhibited no significant difference in cell proliferation and cell cycle distribution. Consistent with the MTT assay and flow cytometry detection data, the colony-forming assay demonstrated that the low concentration of ginsenoside CK (5–15 μM) had little effect on the proliferation activities of HCC-LM3 and SMMC-7721 cells.

Tumor metastasis, one of the characteristics of tumor malignancy that significantly influences tumor progression and prognosis, is accomplished by a series of steps, including angiopoiesis, cell adhesion, degradation of the extracellular matrix (ECM), migration, invasion and transport to other tissues through blood vessels and/or lymphatic vessels [36]. Previous studies have shown that ginsenoside CK could inhibit cell invasion and migration at low toxic concentrations of 1–100 μg/mL in B16-BL6 and HT-1080 cells, while Rb1, the precursor of ginsenoside CK, exhibited little influence on the invasion potential of the two cell lines [27]. Another study described that nontoxic doses of ginsenoside CK significantly decrease MMP-9 expression [37], which plays a major part in tumor invasion and angiogenesis, at both the gene and protein levels in astroglioma cells. Furthermore, ginsenoside CK inhibited MHCC97-H cell adhesion and invasion in vitro dose-dependently [38]. Notably, tumor metastasis was found in the liver when MHCC97-H cells were subcutaneously implanted; in contrast, mice treated with different doses of ginsenoside CK showed a sharp decrease in liver metastatic nodules compared with the control group [38].

Epithelial–mesenchymal transformation (EMT) of tumor cells is imperative for tumor metastasis, marked by a damage of the cell epithelial phenotype and promotion of the cell mesenchymal phenotype [39], which contribute to the cell invasion and migration activities. During EMT, upregulation of N-cadherin and Vimentin and a decrease in the E-cadherin protein level were observed. In addition, the transcription factors involved in EMT, Snail and Twist, are the major inducers in retaining the invasive mesenchymal phenotype [40]. A relationship between hypoxic conditions and EMT in human carcinomas has been found. Hypoxia regulates tumor EMT through various mechanisms, including adjusting EMT signaling pathways, suppressing EMT-TF expression and modulating EMT-associated miRNA and lncRNA networks. In HCC cell lines, HepG2 was identified as epithelial on the basis of the expression of E-cadherin, while SMMC-7721 and HCC-LM3 were considered mesenchymal due to the high expression of N-cadherin and relatively low level of E-cadherin. In line with these results, we observed that the protein expression levels of N-cadherin and Vimentin in HCC-LM3 and SMMC-7721 cells were upregulated under hypoxia, as well as the EMT-TFs Twist1 and Snail, while E-cadherin was downregulated in HepG2 cells, signifying the activation of the EMT process in HCC. The NF-κB signaling has been recognized as a key factor in neoplasia and the progression of inflammation-induced cancers. NF-κB is an oncogene that is related to tumor cell proliferation, metabolic remodeling and EMT [41]. NF-κB signaling could induce the expression of EMT marker proteins and EMT cytokines in direct and indirect patterns. Accumulating studies have demonstrated that overactivation of NF-κB is closely related to hepatitis and hepatocellular carcinoma. In our study, under stimulation with TNFα, which served as an inducer of the NF-κB signaling pathway, the promotion of EMT in HCC cells was found to be the trend of N-cadherin, Vimentin and other EMT-related proteins.

Mounting evidence has indicated the association between HIF-1α and the NF-κB signaling pathway in tumor cells. In line with our study, upregulation of HIF-1α under hypoxic conditions heightened NF-κB signaling, which was demonstrated by the increased binding activity of NF-kB p65 proteins and NF-κB DNA after initiation of hypoxia, and inhibition of NF-κB was associated with the suppression of hypoxia-induced migration in pancreatic cancer cells [42]. In addition, stimulation of NF-κB could stabilize and activate HIF-1α protein and signaling in normoxia [43]. Data in the GEPIA database [44] suggested that the HIF-1α and NF-κB target genes are positively related in HCC tissues, as well as EMT-related proteins and cytokines, while higher HIF-1α expression correlated with worse prognosis for HCC patients. In our study, we confirmed that activating NF-κB signaling could increase HIF-1α and its target transcriptional expression at the gene and protein levels, while CoCl_2_-induced hypoxia also facilitated the phosphorylation of IκB and the nuclear translocation of the NF-κB subunit, which implies the activation of NF-κB signaling, followed by an increase in downstream proteins. In summary, the crosstalk of HIF-1α and NF-κB signaling could be a potential target to improve the therapeutic effect of HCC.

Based on these studies and clinical demands, we detected that the effect of ginsenoside CK on hypoxia-induced EMT results in HCC cells with a different phenotype (epithelium-like HepG2, mesenchymal-like HCC-LM3 and SMMC-7721) in the present study. Ginsenoside CK treatment reversed the EMT process by modulating EMT-related biomarkers in hypoxia- or TNFα-treated cell lines. The expression and stabilization of HIF-1α induced by hypoxia could be destroyed by ginsenoside CK treatment, as shown in our immunoblotting and immunofluorescence staining results, followed by the downregulation of HIF-1α targeted genes, which signified the inhibitory activity of ginsenoside CK on HIF-1α signaling. The results also demonstrate that ginsenoside CK inhibited the hypoxia-induced activation of NF-κB signaling by decreasing the phosphorylation of IκB and p65 translocation. Directly activating NF-κB signaling by treating cell lines with TNFα was also alleviated by ginsenoside CK in a dose-dependent manner, while NF-κB-induced increases in the gene and protein levels of HIF-1α, and the targeted genes could be blocked under ginsenoside CK treatment. MMPs, a large family of proteolytic enzymes, are ion-dependent proteinases capable of cleaving components of the extracellular matrix and contribute to tumor invasion and metastasis. As shown in our present data, ginsenoside CK treatment of two different HCC cells caused a reduction in the expression of MMP2 and MMP9 in a dose-dependent manner. The results were in line with a previous study showing that ginsenoside CK could suppress HCC migration by downregulating the expression of MMP2 and MMP9 in MHCC-97h cells [38]. In vivo studies verified that treatment with ginsenoside CK not only inhibited tumor growth (reduced tumor volume and weight) but also suppressed the local EMT process resulting from the regulation of HIF-1α/NF-κB signaling. The data also displayed smaller tumor necrotic areas in the ginsenoside CK-treated group, indicating a lower degree of tumor malignant progression.

In previous studies, the 26-week repeated-dose oral toxicity of ginsenoside CK was detected with NOAEL 120 mg/kg and 40 mg/kg in female and male rats [45], respectively. Other related works showed that the serum concentration of ginsenoside CK reached the maximum concentration of 4.57 ± 3.16 ng/mL determined using LC-MS/MS analysis in healthy volunteers administered oral red ginseng [46]. However, pharmacokinetic research on ginsenoside CK needs more exploration due to the dependency relationship between biological effects and the bioavailability and metabolism of the therapeutic drug.

## 5. Conclusions

In summary, the research results verify the crosstalk between HIF-1α and NF-κB signaling in HCC. Moreover, pronounced in vitro and in vivo results illustrate that ginsenoside CK, metabolized by intestinal flora with other PPD-type ginsenosides, could reverse EMT in hypoxia-induced HCC by blocking the communication of HIF-1α and the NF-κB signaling pathway. It is attractive to explain that ginsenoside CK may have significance for further exploration as a potential therapeutic method against HCC. Nonetheless, the functional mechanism, pharmacokinetics and therapeutic effect of ginsenoside CK in liver cancer therapy still need further study to provide more accurate valuable evidence for ginsenoside CK in clinical application.

## Data Availability

Not applicable.

## Supplementary Materials

The following are available online at https://www.mdpi.com/article/10.3390/foods10061195/s1, Table S1: Details of the antibodies, Table S2: Primer Sequences, Figure S1: Cytotoxicity of CoCl_2_ treatment on HCC cell lines after 48 h were determined by MTT assay, Figure S2: Effect of ginsenoside CK on metastasis potential of HCC cell lines triggered with TNFα were determined by scratch assay.
