# Supplementary material for: Ginsenoside CK Inhibits Hypoxia-Induced Epithelial–Mesenchymal Transformation through the HIF-1α/NF-κB Feedback Pathway in Hepatocellular Carcinoma
Source: Foods. 2021 May 26;10(6):1195. doi: 10.3390/foods10061195 (PMC8227303; doi:10.3390/foods10061195)
Supplement: Supplementary file 1 [file foods-10-01195-s001.zip › foods-1240505-supplementary.pdf]

Table. S1. Details of the antibodies

Antibody	Source	Catalog Number	Dilution Rate
E-cadherin	Rabbit	20874-1-AP	1:7500
Vimentin	Rabbit	10366-1-AP	1:2500
N-cadherin	Rabbit	22018-1-AP	1:5000
Snail	Rabbit	26183-1-AP	1:1000
HIF-1α	Rabbit	20960-1-AP	1:5000
Twist1	Rabbit	25465-1-AP	1:1000
IκB-α	Rabbit	10268-1-AP	1:2000
p65	Rabbit	66535-1-Ig	1:3000
Lamin B1	Rabbit	12987-1-AP	1:5000
ICAM-1	Rabbit	60299-1-Ig	1:10000
CA9	Rabbit	11071-1-AP	1:2000
c-Myc	Rabbit	10828-1-AP	1:2000
HIF-1α	Mouse	66730-1-Ig	1:5000
VEGF	Mouse	66828-1-Ig	1:2000
phospho-IκB-α	Rabbit	2859	1:1000
phospho-p65	Rabbit	3031	1:1000
MMP-2	Rabbit	CY5189	1:1000
MMP-9	Rabbit	CY1226	1:1000

Table.S2 Primer Sequences

HIF-1α	F	5’-CTCATCAGTTGCCACTTCCACATA-3’
	R	5’-AGCAATTCATCTGTGCTTTCATGTC-3’
VEGF	F	5’-TCACAGGTACAGGGATGAGGACAC-3’
	R	5’-CAAAGCACAGCAATGTCCTGAAG-3’
CA9	F	5’-ACCAGACAGTGATGCTGAGTGCTAA-3’
	R	5’-TCAGCTGTAGCCGAGAGTCACC-3’
c-Myc	F	5’-GGCTCCTGGCAAAAGGTCA-3’
	R	5’-CTGCGTAGTTGTGCTGATGT-3’
ICAM-1	F	5’-AAACACTAGGCCACGCATC-3’
	R	5’-CCCACCACTTCCCCTCT-3’
GAPDH	F	5’-GGGGAAGGTGAAGGTCGGAG-3’
	R	5’-TCTCGCTCCTGGAAGATGGTGAT-3’

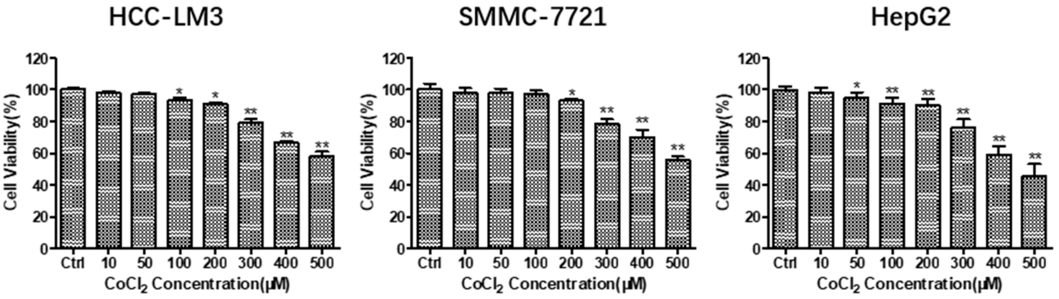

Fig. S1 Cytotoxicity of $CoCl_2$ treatment on HCC cell lines after 48h were determined by MTT assay. Figures are presented as mean±SD of six replicates.

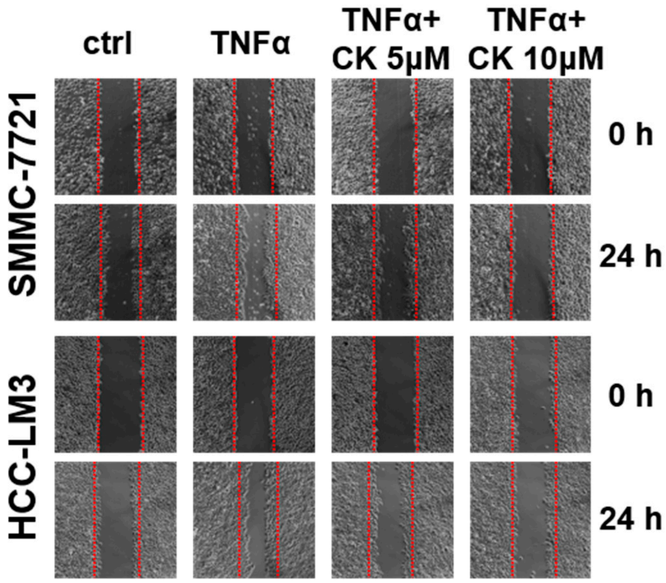

Fig. S2 Effect of ginsenoside CK on metastasis potential of HCC cell lines triggered with TNFα were determined by scratch assay.
